# Editorial: Exploring volatile organic compounds in fruits and flowers: aroma, biosynthesis, and ecological impact

**DOI:** 10.3389/fpls.2025.1701678

**Published:** 2025-09-26

**Authors:** Vikas Dadwal, Deepak Kumar Jha, Mariam Gaid, Robin Joshi

**Affiliations:** ^1^ Vegetable and Fruit Improvement Center, United States Department of Agriculture (USDA) National Center of Excellence, Department of Horticultural Sciences, Texas A&M University, College Station, TX, United States; ^2^ Institute of Microbiology, University Greifswald, Greifswald, Germany; ^3^ Perelman School of Medicine, Institute of Translation Medicine for Therapeutics (ITMAT), University of Pennsylvania, Philadelphia, PA, United States

**Keywords:** phenylpropanoids, fatty acid derivatives, metabolomics, volatilomics, transcriptomics, volatile organic compounds (VOCs), aroma

Volatile organic compounds (VOCs) emitted by fruits and flowers play an important role in plant environment interaction through their distinctive aromas and signaling properties. These compounds, primarily terpenoids, phenylpropanoids, and fatty acid derivatives, are synthesized *via* complex metabolic pathways influenced by genetic, developmental, and environmental factors in fruits and flowers. Additionally, VOCs contribute significantly to plant reproductive success by attracting pollinators and seed dispersers, while also serving defensive roles against herbivores and pathogens. Metabolomics and other omics techniques offer a comprehensive approach to profiling and quantifying VOCs in plants and fruits, enabling deeper insights into their biosynthesis, regulation, and ecological functions. Understanding these VOCs not only supplements our understanding of plant biology but also has applications in agriculture, food science, and the fragrance industries. Therefore, invitations were sent to outstanding experts in this field to contribute their manuscripts to this Research Topic. An effort was undertaken to investigate metabolites, their diversity, and underlying regulatory mechanisms through an integrated omics approach. Finally, a total of 94 authors contributed, and 12 research articles were published in the present Research Topic.

Consumers’ perception of longan fruit is heavily influenced by aroma, a key factor affecting flavor quality. In a recent study by Hu et al., a high-density linkage map was created through SNP genotyping, followed by QTL mapping to identify genes responsible for aromatic traits. Using the constructed high-density Bin map and validation through collinearity analysis, fifty-six QTLs were identified for nine aroma-related characteristics, including (E)-2-hexenal, ethyl acetate, ethyl butyrate, ethyl crotonate, and total ester content. Further analysis revealed that six candidate genes control ester biosynthesis. This study offers valuable insights for breeders working on high-quality aromatic fruit crops. Besides genetic makeup, the fruit metabolome also plays a significant role in its flavor profile. A recent study by Li et al. demonstrated this through comprehensive metabolite analysis of kumquat fruits. Sucrose and limonin are key factors contributing to sweetness and bitterness, respectively. The red color of the fruit peel is linked to the upregulation of anthocyanins and carotenoids. Screening 1719 metabolites showed that the peel is rich in flavonoids, while the seeds contain bioactive triterpenoids like limonin. Considering health benefits, a study by Sonigra and Meena found that essential oils (EOs) from *Cymbopogon martinii* (Roxb.) Wats. have both aromatic and bioactive properties. Notably, EOs were analyzed at three developmental stages through hydro-distillation combined with GC-MS profiling. The analysis identified 59 compounds, with the highest amounts during the reproductive stage with an increased oil yield. This significant yield further demonstrated antibacterial and antifungal activities. Additionally, the post-reproductive stage showed higher antioxidant activity. This study effectively highlights the importance of harvesting time for maximizing the bioactive EO yield, which is crucial for future therapeutic and industrial applications.

In addition to being primary contributors to aroma, volatiles, particularly EOs, play key roles in plant defense and exhibit antimicrobial, antioxidant, antifungal, and insecticidal properties, making them valuable in food preservation, medicine, and pest management. Peng et al. explored the volatile diversity at different developmental stages and their potential antimicrobial roles. in *Zanthoxylum armatum*, an aromatic Rutaceae shrub, especially rich in bioactive EOs concentrated in its pericarp, showed strong antibacterial, antioxidant, and anti-inflammatory effects. The systematic analysis of *Z. armatum* fruits across seven growth stages and the resulting volatile fingerprint mapping, revealed stage-specific EO profiles dominated by linalool, D-limonene, and sabinene. Early stages accumulated simple monoterpenes, whereas alcohols and esters increased with maturation, with peak abundance in July–August, identified as the optimal harvest period. The bioassays of these EOs confirmed strong insect-repellent effects against *Tribolium castaneum* (without lethal toxicity) and significant antifungal activity against *Corynespora cassiicola*, particularly at the mid-July (mature) stage. Activities appeared driven by synergistic compound interactions rather than single constituents. Peng et al. findings provide the first systematic temporal profile of *Z. armatum* EOs, linking harvest stage to bioactivity and offering a basis for developing eco-friendly insect-repellent and antifungal agents.

Jujube flowers, abundant in diverse volatile compounds, enhance sensory appeal and stand out among aromatic plants. Ren et al. exhaustively performed sensory evaluation in jujube flowers across developmental stages and varieties, and compared with other aromatic plant flowers. A total of 65 VOCs were identified, and aroma fingerprinting revealed 24 distinct aromas, 14 of which intensified from bud stage to full bloom. Varieties such as Fuxiang, Dongzao, and Xingguang exhibited significantly stronger aromas at the flowering stage than others. Sensory evaluation further indicated a male preference for the fragrance of jujube flowers. Their finding, also highlighted the richness in volatile diversity and abundance, has potential for spice development, and could be a basis for future research on floral-derived spices.

While the effects of genetics, environment, and physiological status on crop volatile composition have been extensively studied, the contribution of microbial communities to shaping these volatile profiles has received far less recognition. Jia et al. integrated untargeted metabolomics and metagenomics to investigate the role of plant–microbe interactions in shaping the aroma profile of flue-cured tobacco leaves. Comparative analyses between light aromatic tobacco (LAT) and strong aromatic tobacco (SAT) detailed that LAT contained significantly higher sugar metabolites (e.g., sucrose, fructose, maltose), while SAT was enriched in acids and amino acids (e.g., xylonic acid, tartaric acid, L-phenylalanine). Metagenomic analyses demonstrated that LAT when dominated by *Methylorubrum*, *Pseudomonas*, and *Pseudokineococcus*, promoted sugar accumulation, whereas SAT when enriched in *Methylobacterium* and *Sphingomonas*, enhanced acid and amino acid metabolism but inhibited sugar accumulation. Redundancy analysis highlighted the bidirectional regulatory role of *Pseudokineococcus*, emphasizing complex microbe–metabolite interactions in shaping aroma profiles. It was worth noting that the conserved microbial functional traits modulate host metabolism, offering physiological guidance for tobacco aroma regulation and broader strategies for microbiome-mediated quality improvement in crops.

Recent advances highlight the complex metabolism and genetic regulation underlying volatile-mediated floral and fruit traits in grapes and pear. Darwish et al. provided the first comprehensive volatilomic characterization of *Muscadinia rotundifolia* flowers, identifying 150 volatiles across 16 genotypes. Multivariate analyses distinguished female and perfect flowers by 11 diagnostic terpenes. Perfect flowers emitted higher terpene quantities, aligning with greater pollinator attraction, while female flowers suffered from lower volatile emission and floral abnormalities, explaining reduced fruit set unless assisted by controlled pollination strategies. Similarly, Wu et al. characterized aroma diversity in six grape cultivars with contrasting aroma types. Muscat cultivars accumulated monoterpenes, the strawberry-type was defined by ester biosynthesis, and neutral types enriched C_6_/C_9_ compounds, preceded by the high expression of responsible genes in each case. Co-expression network analysis revealed distinct modules related to alcohols, carbonyls, fatty acids, and monoterpenes. Within the same context, aroma differentiation in pear was investigated by Wang et al. by integrating metabolite profiling with transcriptome analysis across three *Pyrus communis* cultivars rich in aroma and three *Pyrus pyrifolia* cultivars known to lack aroma. Among 510 identified volatiles, 16 ester and alcohol compounds were enriched in *Pyrus communis* relative to aroma-deficient *P. pyrifolia*. The existence of these metabolites was linked to amino acid degradation pathways, where key differences could be attributed to the activity of related genes encoding monoacylglycerol lipase, threonine dehydratase, and acyl-CoA dehydrogenase. Collectively, these studies provide mechanistic insights into aroma and floral volatiles, offering targets for breeding and metabolic engineering to enhance aroma quality, pollination efficiency, and ultimately, crop quality. Wang et al. described the integrated volatile metabolite and transcriptome analyses, which provide insights into the warm aroma formation elicited by methyl jasmonate in carrot roots. The results identified 1,227 volatile organic compounds and 972 differentially accumulated metabolites, along with 4,787 differentially expressed genes. This study enabled an understanding of MeJA-mediated aroma formation in carrots. Qian et al. described the role of volatiles on insect behavior in *Aquilaria sinensis*. A total of 11 volatile compounds with electrophysiological activities were detected. These volatile components can be considered as an alternative to chemical insecticides in pest control. Another investigation by He et al. revealed the mechanism of volatile alterations during fruit development of ‘Ehime 38’ (*Citrus reticulata*) and its bud mutant. A total of 35 volatile compounds were identified in the pulps of both WT and MT across five developmental stages. Most of the genes in the MEP pathway showed a positive correlation with volatile levels, as determined by transcriptomic and RT-qPCR studies. A detailed metabolomics and transcriptomics study will be valuable for fruit flavor development in citrus.

In conclusion, the Research Topic exhaustively details the critical roles of volatile compounds in shaping the aroma, flavor, and bioactive properties of fruits, flowers, and other plant organs. It also highlights that the aroma profiles are influenced by a complex interplay of genetics, developmental stage, metabolite composition, and plant-microbe interactions, with specific volatiles such as monoterpenes, esters, and alcohols often driving sensory appeal, pollinator attraction, and defense responses. Integrative approach combining metabolomics, transcriptomics, and sensory evaluation revealed stage- and genotype-specific accumulation patterns, identified key biosynthetic genes, and linked volatiles to biological activities such as antimicrobial, antifungal, and insect-repellent effects. The insights in this Research Topic provide a foundation for breeding, metabolic engineering, and the development of eco-friendly plant-based products, ranging from high-quality aromatic fruits and floral spices to natural pest management and therapeutic agents.

